# An Inquiry into the Functions of the Spleen, Liver, Pancreas, and Thyroid Gland

**Published:** 1806-09

**Authors:** Benjamin Rush

**Affiliations:** Professor of the Institutes and Practice of Medicine, in the University of Pennsylvania


### Medical and Phyfical Journal.

VOL. XVI.] SEPTEMBER, 1806. [NO. 91.

*Printed for* R. PHILLIPS, *by* W. Thorne, *Red Lion Court, Fleet Street, London.*

Dr. JARDINE having received the following Communication from his friend Dr. RUSH, of Philadelphia, requests the Editors of the Medical and Physical Journal will insert it in their next Number.

*Liverpool, July* 21, 1806.

### *An Inquiry into the Functions of the Spleen, Liver, Pancreas, and Thyroid Gland; by* BENJAMIN RUSH, M. D. *Professor of the Institutes and Practice of Medicine, in the University of Pennsylvania.*

### OF THE FUNCTION OF THE SPLEEN.

FOUR uses have been ascribed to the spleen. It has been said to prepare the blood for the secretion of bile from it by the liver; to be the organ in which the red globules of the blood are formed; to be a counterweight to that of the liver, on the left side; and lastly, to afford an occasional supply of blood to the vessels of the stomach which secrete the gastric liquor, when that viscus is unduly distended with food. If the use I am about to ascribe to the spleen be admitted, it will be unnecessary to state objections to the opinions which have been mentioned.

I shall begin this inquiry by delivering the following proposition.

All the motions which go forward in the human body are produced by external and internal stimuli. These stimuli exert their influence directly, or indirectly, upon the blood-vessels. From innumerable causes, they are liable to become *excessive* in their force. Such is the excess of this force, and such the frequency of its occurrence from exercise, labour, intemperance, passions of the mind, and disease, that a provision to defend the tender and vital parts of the body from the effects of this force, seems to be a necessary appendage to the body. This provision, I believe, to be the SPLEEN. My reasons for this opinion are founded upon the following facts.

1. The *structure* of the spleen. It contains but one artery, and that a large one, with veins that ramify through every part of it. This artery is larger than that of the liver, which is four times as large as the spleen. It is moreover, stronger, according to Dr. Wintringham, than the aorta, from which it is derived, in the ratio of 1212 to 1000. Its veins possess a peculiar and specific strength, which assists in performing the office I have assigned to it. In having but one artery, it differs from those viscera, which have certain offices to perform upon the blood. These viscera are the lungs, the liver, and the heart, each of which has an artery intended for the exclusive purpose of its nourishment. The spleen having no work to perform upon the blood, and serving no other purpose than a temporary reservoir for it, is nourished by its single artery. Another artery would have been superfluous to it. It has, moreover, no excretory duct. Its texture is soft and spongy, and of so distensible a nature, that it will admit of an increase in its weight from blood to three or four pounds, according to Dr. Baillie, without discovering the least mark of a departure from its natural state, in which it contains about a pound of blood.* It is from the facility with which it is distended, that it has been compared to the corpora cavernosa penis.

2. The *situation* of the spleen. It is placed near the heart, the centre and prime mover of inordinate and violent motions in the blood-vessels, and in a part of the belly, in which from the frequent and lax state of the stomach and bowels, and from the disproportionate space it occupies, to its size, it is capable of more prompt and greater distention, than it could have had in any other part of the body. To enable it always to retain its distensible power, it is never lessened in its size by fat.

3. The

---

* In an account of a dissection, communicated to Dr. Duncan, by Mr. James Elliot, he says, "the Spleen was found, when taken from the body, to weigh eleven pounds thirteen ounces," and that its texture, figure, and bluish colour, were little, if at all, changed." *Medical Commentaries.*

3. The phenomena which take place in several of the common exercises of life. Where is the school-boy who after running for fifteen or twenty minutes, in the ordinary plays of schools, has not felt a pain in the left side? This is so great sometimes as to compel him to sit down half bent, and even to make him cry out for relief. The name of this pain, I well recollect, indicated its cause. It was the "spleen." In laughing, the spleen performs the same kind office of opening a waste gate, for the torrent of blood excited into action, by the violent and excessive agitation of the blood-vessels; hence we so often observe persons in paroxysms of laughter, press upon the left side, to lessen the pain produced by the distension of the spleen, and hence too, the phrase of "splitting the sides with laughing." It is the left side only that is in danger of bursting, and the spleen only, in this side. When sudden death occurs from laughing, it is probably induced by a rupture of the spleen, an accident which has, now and then, dissections teach us, occurred from other causes. The pain in the left side, which is felt in riding a hard-trotting horse, appears like that which is induced by running, and laughing, to arise wholly from an undue distention of the spleen. In all these cases, it performs the office of a bason, held by the hand of nature to receive, for a while, several pounds of blood, in order to preserve the system from disease and death. It is only when the spleen is distended to the extent of its capacity of retaining blood, that it imparts a sense of pain, for I shall say presently, it possesses but little sensibility in proportion to the number of its nerves.

4. The *quality* of the blood which has been procured from it. It is less coagulable than blood obtained from the arm by bleeding, or discharged from wounds. I have ascribed this to the force with which it is thrown into the spleen, or to the feeble action of the veins, in propelling it back again into the circulation, both of which we know impair the coagulable quality in the blood. I am aware this quality in the blood is denied by Dr. Saunders, but he opposes to it only a single experiment, made under circumstances which do not contradict the numerous experiments of several other, equally respectable physiologists.

5. The nature of the diseases which produce obstructions in the spleen. These are fevers of all kinds. Dr. Jackson says, that in all the soldiers whom he opened, who had died of the yellow fever at St. Domingo, he found the "spleen full, and ready to burst, or lax, and filled with grumous blood." In nearly an hundred persons who died of the bilious tertians of Minorca, whose bodies Dr. Cleghorn examined after death, he found the spleen "large, sometimes weighing four or five pounds, and so excessively soft and rotten, that it had more the appearance of congealed blood, wrapped up in a membrane, than of an organical part." The same excellent physician tells us, that in a number of persons who died of the dysentery, he found the spleen in most of them "more or less in a putrid condition," induced probably in both cases, by the great force or quantity of blood thrown into it by those two diseases. Morgagni says, "an enlargement and obstructions in the spleen are generally found in persons who die of chronic fevers." The excitement of the blood-vessels by the undue exercises of the faculties of the mind, produces the same morbid affections of the spleen. This has been proved by Dr. Prost, who found it diseased in seventeen out of eighteen maniacs, whose bodies he examined after death. The effects of the malignant passions, in a more especial manner in disordering the spleen, are admitted in common conversation, by our calling a *malicious*, a *splenetic* man. It has been remarked, that in persons who die suddenly, the spleen is found to be of its natural size. The sudden destruction of the excitement of the blood-vessels in these cases, does not give the spleen time enough to open its friendly door, to receive the excess of the stimulus which thus destroys life, by abstracting from them for a while, three or four pounds of blood.

6. The disease which most commonly follows an enlarged, or obstructed spleen, and that is hemorrhage. Hippocrates long ago ascribed a bleeding from the nose, to an obstruction of the spleen. Vanswieten says, he once attended a patient, in whom an uneasiness and tension of the spleen, enabled him to predict a return of the same disease. Lieutaud mentions nine cases of persons dying with hemorrhagies, in whom the spleen was found after death, diseased from enlargement, and what he calls putrefaction. Four of those hemorrhagies were from the nose, the others were the stomach, bowels, and hemorrhoidal vessels. There are few physicians that cannot subscribe to the connection between hemorrhagies and obstructions of the spleen. I think I have observed them most frequently to occur from the liver and stomach.

7thly and lastly. The diseases which follow the loss of the the spleen, whether by accident or design, in men, and other animals. These are an enlargement of the liver, flatulency, indigestion, head-ach, and an increased secretion of saliva, urine, and semen.

I might here take notice of the existence of a spleen, in all classes of animals, as a proof of its being intended for the purpose I have mentioned. I might likewise point out the difference of the spleen in the human species, from that of brutes, consisting chiefly in a texture calculated to afford more promptly and freely, a receptacle for the blood, when exerted into tumultuous motions; but this would lead me from the subject of the present inquiry. I have supposed this difference, so favourable to the quick distension and capacity of the spleen in the human species, to have been necessary, in consequence of our blood-vessels being more exposed to excessive motions than those of inferior animals, through the medium of our greater portion of mind.

How far the spleen may act by absorbing and suffocating undue impressions upon the nervous system and the mind, I know not; but I think it probable, it serves this important use in the animal economy. My reasons for this opinion are founded upon the great number of nerves which belong to the spleen, compared with its size, and upon its feeble sensibility. It is seldom inflamed, and when it is, the inflammation is attended with but little pain. Even wounds of the spleen are painful in a very feeble degree.

Upon taking a view of the manner in which other parts of the body are guarded from the evils of excess in quantity and motion, we shall be more readily induced to admit the facts and reasonings, in favour of the use of the spleen, which I have mentioned. The eye is kindly defended from the evils of redundant light, by its black pigment; the liver from redundant bile, by a gall-bladder; the blood from redundant oil, by cells; and the numerous cavities of the body, from redundant lymph, by a system of absorbents, happily calculated for that purpose. Why should not the blood-vessels possess a similar advantage to protect themselves from destruction, by means of a temporary reservoir of their redundant motions, or quantity of blood? They would have been imperfect without it; and were it possible for the brain, the liver, the stomach, and the bowels to express an opinion upon this subject, I believe they would say, they owed the preservation of their blood-vessels from rupture and obstructions, and of their nerves from disease in a thousand instances in the course of an ordinary life, to the prompt and friendly offices of the spleen; and were it possible for this long-degraded, and ever-insulted viscus, to obey the call to inanimate nature to praise its Creator, I believe it would raise as high a note in honour of his wisdom and goodness as the eye, or the ear, or any other noble and obviously useful part of the human body.

I shall dismiss this physiological view of the function of the spleen, by briefly hinting at its application to pathology, and the practice of physic.

1. We are taught from the use that has been assigned to it, the necessity of blood-letting in all those diseases in which it has been found to be unduly distended with blood. Death is probably, in these cases, the effect of an inability in the spleen to afford a reservoir for a quantity of blood, sufficient to lessen the inordinate motions excited by it in the system.

2. We are led by the opinion that has been delivered, to suspect obstructions to exist in the spleen, in all habitual hemorrhages, more especially when they occur from the stomach, liver and nose, and to rely not less upon astringent, than deobstruent medicines, in our attempts to cure them. I am happy to find there are precedents in favour of this practice. Donatus and Vogel, mention cases of a vomiting of blood being cured by removing obstructions of the spleen.

3. The use that has been assigned to this viscus, should lead us to suspect that many of the diseases of the liver, stomach, lungs, and brain, are the effects of obstructions in the spleen, and to employ suitable remedies to remove them. I am the more disposed to inculcate this suspicion from a fact related by Dr. Prost, in his excellent work, entitled "Médecine eclairée par l'observation, et l'ouverture des corps." He says, of thirty-eight persons who died of pulmonary consumption, he found the spleen enlarged from two to six times its natural size, in one half of them. This is probably often the case when that disease is attended with a spitting of blood. It is possible, the efficacy of a salivation in curing it, may depend in part, upon its restoring the natural and healthy function of the spleen.

### Of the Function of the Liver.

The design of the liver, I believe to be, to receive the blood from every part of the body, in order to subject that part part of it, which had not been completely animalised, or divested of its chylous properties, to a secretory process, and afterwards to pour the product of this secretion, mixed with the liquor of the pancreas, into the duodenum, to be absorbed, or otherwise taken up by the lacteals, and conveyed with the chyle from the stomach into the blood-vessels, in order to be completely converted into red blood, for the purpose of serving the various and important uses, for which that fluid is intended in the human body.

The facts which have led me to adopt this opinion, are as follow.

1. The liver is present in nearly all animals. In this respect, it is upon a footing with the stomach. Of course there is reason to suppose it is designed to perform an office in the system, equally necessary with the stomach, to the support of life. It is no objection to the truth of this remark, that in some animals there is no gall-bladder attached to the liver, and that in others, it discharges its contents into the stomach, or the bowels remote from the liver; for I hope to shew presently, that the cystic and hepatic bile, serve very different purposes in the animal economy.

2. The immense and disproportionate size of the liver, in the fœtus, compared with its size in adults, the design of which appears to be, that nourishment may be carried on exclusively by that viscus, without any aid from the stomach.

3. The size of the liver in adults, and the quantity of bile which is secreted, supposed by Dr. Haller to be four and twenty ounces in four and twenty hours; five-sixths of which he supposes, pass directly into the duodenum. It is not probable this large apparatus, and copious secretion, can be of an excrementitious nature, and I shall hereafter mention some facts to prove, that it is not essential to the production of the chyle that descends from the stomach, and that it is effused, subsequent to the chyle having passed by the duodenum.

4. Chyle is found in the blood, after it has passed through the lungs. This has often been observed, when it has been drawn, soon after a full meal. It has likewise been demonstrated by Dr. Hutchinson, in his inaugural dissertation, upon "the conversion of chyle into the blood," published in the year 1804, by a number of experiments, made upon the blood of living dogs. The chyle in these cases, partakes too much of the nature of the aliments from which it is formed to be changed into blood in the lungs, by the action of the air upon it, without undergoing a second chylopoetic process in the liver.

5. The quality of the venous blood, from which the hepatic bile is secreted. It is less disposed to putrefaction than arterial blood, taken from any other part of the body. This has been ascribed by Dr. Caldwell, to its having parted with its oxygen. It is possible this may be one of the causes of its being less putrescent than arterial blood; but I am disposed to ascribe it chiefly to its containing a quantity of imperfect chyle, which we know passes more slowly into a putrid state than blood. That this is the case, has been proved accidentally by Dr. Hutchinson, by an experiment made without any reference to this subject, or even to that of his dissertation; also by an experiment, made by Dr. Rollo, in which he found the blood of a man in a diabetes, to be less putrescent than the blood of a person in health. In that disease, we know the blood contains a preternatural quantity of chyle, often so great, as to discover itself, not only in the urine, but in several other of the secretions. Dr. Haller says, he has seen fat in the vena portarum. I suspect the matter he supposed to be fat, was a portion of imperfect chyle.

6. The quality of the hepatic bile. Dr. Boerhaave, who says, he had tasted it, asserts that it is "mild, sweetish, and watery. Dr. Haller says of it, "dulcior hepatica bilis, cystica amara;" but he adds, that he found a considerable bitterness in it, in a man who had been hung, and in a woman who had died suddenly. In both cases, this bitterness was probably imparted to it in the act of dying, by a mixture of cystic bile with it. In animals, which have no gall-bladder, the Doctor admits the hepatic bile to be "uniformly sweet." It is certain the livers of those animals, which form a part of our aliment, have not the least bitter taste, except in those cases in which the contents of the gall-bladder have accidentally fallen upon them, in the act of dressing them for the table. The hepatic bile is always sweet in new-born infants. By a chemical analysis, even the cystic bile of an adult, yields a portion of albumen, which we know to be one of the component parts of the chyle.

7. There are several experiments related by Dr. Fordyce, which prove chyle to be formed by the action of the saliva and gastric juice upon the aliment, without the mixture of hepatic bile with it. The Doctor tied up the ductus communis of an animal, and found the chyle afterwards to possess its usual healthy properties. The same healthy healthy and natural state of the chyle has been observed in those cases, when, not only the excretion, but the secretion of bile has been suspended by that torpid state of the liver, which has lately received from Dr. Pierson, the appropriate name of Hepatalgia.

8. The structure, situation, and function of the PANCREAS. It resembles the salivary glands in its structure; it secretes a liquor, which possesses the same dissolving and animalizing properties as the saliva, and it pours this liquor so directly upon the hepatic bile, in the common duct, before it enters the duodenum, as to act upon it in a concentrated state, and thereby to change it into perfect chyle. By ascribing this use to the Pancreas, we rescue it from its insignificant office of performing a work of supererogation only to the saliva and gastric juice, and give it a little sovereignty or independent jurisdiction in the animal economy.

9thly and lastly. I infer, that a second chylopoetic process goes forward in the liver, from the effects of intemperance upon it. It increases its labour, and thereby increases its size. This has often been observed in full feeders. But it does more. It produces a preternatural secretion of bile, more especially when aliments consist of an undue proportion of fat substances, which resist the powers of digestion in the stomach. When this labour has been long imposed upon the liver, we observe it to succumb like the stomach uuder hard usage, and to produce, with its diseases, the same morbid affections in other parts of the body.

Let us next inquire into the use of the gall-bladder, and the cystic bile.

From the situation of the gall-bladder, from the acute angle its duct makes with the hepatic duct, where they form the ductus communis, and from the circumstances which influence its fulness and depletion, I believe it to be intended wholly to serve the same purpose with respect to the liver, which the spleen serves to the whole sanguiferous system, that is, to afford a receptacle for redundant bile, and thereby to prevent the obstruction of the hepatic bile into the duodenum, and its regurgitation into the pori biliarii. Unless this provision had been made for an excess in the secretion of bile, to which so many accidental causes contribute, the liver would have been exposed to disease and disorganization every day. It is possible, the less nutritious particles of the hepatic bile may be thrown into the gall-bladder; but whether this be the case, or not, the bile bile appears to undergo a putrefactive process in it. I infer this, from the bitterness it acquires during its stagnation in the gall-bladder. The same bitter quality is the offspring of the putrefaction of certain vegetables, and of some animal substances. It probably takes place in other secretions of the body. Dr. Darwin ascribes the bitter taste we often perceive upon the tongue, to a morbid change in the quality of the liquor, which is secreted upon its surface; and perhaps the bitterness of the wax, which is secreted in the meatus auditorius is derived from a change induced in it, by its stagnation, similar to that which occurs in the bile. It is true, the bile is less putrescent than the blood, according to an experiment made by Dr. Saunders, and since repeated by a graduate, in the University of Pennsylvania; but the same indisposition to putrefaction takes place in certain vegetables, after they acquire, by decay, a bitter taste. Of the truth of this remark, I satisfied myself by the following experiment. Into a two-ounce phial, I put half an ounce of beef, cut into small pieces, with the same quantity of the rotten and bitter part of an apple, and an ounce of water. Into another phial, I put the same quantity of beef and water, and placed them both under the same circumstances of heat. The latter putrefied many hours before the former. Had the rotten part of the apple been exposed alone to heat and air, I have no doubt it would have undergone another putrefaction. The same remark applies to the bile, out of the body. Even the fæces undergo a similar change, and hence we find them inoffensive, as remote causes of disease, unless they have been exposed long enough to the action of the sun and air, to excite in them a second putrefaction. While I suppose the bile to be of an excrementitious nature, I admit that it serves several valuable purposes in the animal economy. In stimulating the bowels, it enables them to propel their contents downwards, and imparts to them at the same time a tone, which is communicated to the whole system. It serves likewise, to precipitate those parts which are incapable of affording nourishment to the body from the chyle; and perhaps, it retards its tendency to putrefaction, especially in hot weather. Should the bile exert this antiseptic quality, it will only be upon a footing with the nitrate of potass and ammonia; both of which, although the offspring of putrefaction, have a powerful effect in preventing it.

The liver has been called, by some physiologists, a secretory, and by others, an excretory organ. The reader will perceive, perceive, that I adopt both these opinions. The hepatic duct discharges its secretion, and the cystic duct its excretion into the duodenum. The gall-bladder appears to be to the liver, what the colon and rectum are to the stomach; the receptacle only of hepatic fæces. The quantities of each, accord with the excess or deficiency of aliment taken into one, and of imperfect chyle received and secreted by the other.

From a review of the function of the liver, which has been delivered, we are led, in the first place, to admire the goodness of the Creator of our bodies, in thus providing two viscera for preparing the matter, which is necessary to repair their daily waste, each of which is provided with an ample apparatus for that purpose. The nourishment of the body is, in this respect, upon a footing with the double senses of vision and hearing. In cases of sickness, indigestion, or long fasting, in which the office of the stomach is suspended, the liver performs a vicarious duty; and when its chylopoetic office is impaired, or interrupted, the stomach, by performing its functions with double care, prevents the evils of an abstraction of nourishment from the body. But there is another instance of alternation between the stomach and liver, in their respective offices. While the former is most busy, the latter, from the pressure of the stomach upon it, is most idle. It is only after the chyle from the stomach has passed by the duodenum, that the liver pours its chyle into it. In this way the bowels and the lacteals are kept in a constant state of moderate tension and activity. The same distention and pressure of the stomach, which impedes the secretion of hepatic chyle, squeezes the cystic bile from the gall-bladder; by which means it is enabled to perform one of the offices I have ascribed to it: namely, to separate the fæcal matters from the chyle. From the situation of the gall-bladder in the concave part of the liver, the pressure of the stomach is necessary to elevate its bile to the level of the cystic duct, in an erect posture of the body. In a recumbent posture, the bile descends more easily into the duodenum, especially in lying upon the back. It is from its more copious effusion into the bowels during sleep, that the practice of going to stool in the morning, has been one of the habits of the human body in all ages and countries. It is from the same cause that persons who are afflicted with an excessive secretion, and excretion of bile, are most apt to be sick, or puke bile in the morning.

I am aware that I differ from all modern and ancient physiologists, physiologists, in supposing that the pressure of the stomach upon the liver, suspends the secretion of bile. I infer it, because pressure suspends secretion in all other glands. We often see it in the kidneys from the pressure of the colon, when distended with fæces upon them. The current opinion of the secretion of bile being increased by the pressure of the stomach upon the liver, took its rise in a belief that its mixture with the aliment in the duodenum, was necessary to constitute chyle. The reader will perceive further, that I do not admit of the gall-bladder being filled by the regurgitation of hepatic bile, in consequence of an obstruction induced upon the ductus communis by the distention of the duodenum. I suppose it to be filled only when the liver secretes and pours its bile in too large quantities to be conveyed into the bowels. In this case, it affords the same relief to the liver, that the spleen affords to the blood-vessels, by becoming its waste-gate. Perhaps the passage of the bile into the gall-bladder, may be assisted by the fibres of the muscular coat of the cystic duct, performing their actions in that direction. In this way we know many fluids are propelled, contrary to their gravity, in different parts of the body.

2. The economy of the double process which has been described for preparing the nourishment of the body, deserves our notice. Had it been otherwise, all that portion of chyle, which was incapable of being made into blood, by passing through the lungs, would have been lost to the system. If the office I have assigned to the liver, be correct, it may be compared to the second bolting cloth of a mill; or perhaps, more properly, a frugal housewife, who collects all the meat which has refused to yield its nourishment to a single operation of fire; and by cooking it a second time in other vessels, and by other modes of heat, extracts from it a second portion of nourishment.

3. From the opinion of the use of the liver which has been delivered, we are enabled to understand the reason, why we often observe large and offensive fæcal discharges, in persons who have eaten little or no food. I have frequently seen them with surprise in children, while labouring under the colera infantum, who had passed many days without retaining a spoonful of aliment, or even of water, upon their stomachs. The same remark is made by Dr. Cleghorn, in his account of the dysentery at Minorca. It is so pertinent to our subject, that I shall insert it in his own words. After mentioning the exhibition of purges, he says, "They brought off a prodigious quantity of round hard hard fœtid lumps, to the great relief of the patient; nor is it easy to conceive how so much had been collected, or where it had been lodged so long; as in some cases I have observed the patients have eaten nothing for two or three weeks, that could furnish much excrement; and during that time had taken several glysters and common cathartics, which brought away liquid stools." From the disposition which the bile has to form itself into solid substances, or what are called gall-stones, I am disposed to believe that the lumps taken notice of by Dr. Cleghorn, or what we call Sybala, are always composed of hepatic fæces. The meconium of children appears to be formed from them.

4. The use which has been ascribed to the liver, leads us to discover the reason why a diseased state of the stomach is so often followed by a diseased state of the liver. When the former performs its work of digestion imperfectly, it imposes double labour upon the latter, in preparing the chyle for the nourishment of the body, and this necessarily brings on disease.

5. It has often been remarked, that the digestive process in the stomach required a healthy and tranquil state of the whole system. The same thing may be said of the chylopoetic process in the liver. It is impaired by all general, and by many local diseases. It suffers, according to Dr. Mosely, in all the acute and chronic diseases of the West Indies. Dr. Paley ascribes to it, the same sympathy with all the diseases of the East Indies. Dr. Boerhaave supposed it to be affected, in ninety-nine out of a hundred chronic diseases, from all their causes. There is scarcely a disease in the head, so light, that does not disturb its natural actions in a greater or less degree.

6. Children are often affected with a sudden swelling and hardness of their bellies, which as suddenly subside from the operation of a purge, or from friction with the hand. It has been ascribed to wind, also to worms in their bowels. May it not be occasioned by a temporary engorgement of the liver, brought on by an excess of aliment taken into the stomach, and by a redundant quantity of chyle prepared from it?

The opinion that has been delivered of the function of the liver, is calculated not only to explain the causes and phænomena of several diseases, but to suggest several inferences that may be useful in the practice of physic.

1. Has the liver been elevated from its former humble and tributary situation in the system, to share with the stomach stomach in the high and important office of preparing nourishment for the body? Let us never forget to inquire into its state, with the same solicitude that we inquire into the state of the stomach in all general diseases.

2. Has a sympathy been pointed out between the stomach and liver in their respective offices? Let us suspect their diseases to be reciprocal. The influence of the liver, when diseased, in bringing on stomach complaints, is well known; and Dr. Thomas Clark has informed us, in his "Observations upon the Diseases of the West and East Inies," that a diseased stomach is the constant, and often the first, sign of a diseased liver in that country.

3. Do we wish to reduce the system by the *sudden* abstraction of nourishment? In vain shall we attempt to do so, by limiting the quantity of aliment taken into the stomach, while the liver continues to supply the lacteals with alimentary matter. It can only be effected by combining active purges with abstinence from food.

4. Do we often meet with a preternatural secretion and excretion of bile appearing in headach, languor, sickness, puking of bile, and an occasional diarrhœa? I have taken the liberty to call this disease, a diabetes of the liver, and have supposed it to depend upon original debility, and increased morbid action in its secretory and excretory vessels. It occurs in the gout, in persons habitually intemperate, and in the season of bilious fevers in all warm countries. It yields to evacuating or tonic remedies, according to the state of the system.

5. Do we now and then meet with a bilious diarrhœa, attended with the appetite and digestion unimpaired? I have called this disease a lientery of the liver, and have supposed that the chyle passes through it into the bowels, without undergoing the change that is necessary to procure its admission into the lacteals. The remedy in this disease is depletion, more especially by abstracting nourishing aliment. I have known it to yield to a diet consisting wholly of vegetables, after purges and the most powerful astringents had been used to no purpose.

6. Does the formation of sound and healthy blood depend upon the healthy action of the liver? and does the liver suffer, more or less, from all general and many local diseases? It follows, of course, that a medicine which is said to act specifically upon glandular parts of the body, and which is known to act powerfully upon the liver, occupies deservedly the first rank of all the articles in the Materia Medica. Such a medicine is mercury, and hence its its usefulness and fame in all general and chronic diseases.

7. Is the stomach, from disease, unable to retain or digest aliment, or is its passage into the stomach, or duodenum, obstructed by schirrus, or any other cause? The use that has been assigned to the liver, should lead us to double our exertions in conveying aliment into the system, by means of injections into the lower bowels. It has been objected to this mode of nourishing the body, that aliment, when absorbed, could not perform that office without being converted into chyle, and that this liquor was prepared exclusively by the stomach. We now see a change of aliment into chyle may be performed, independently of the stomach, by means of the liver.

### Of the Functions of the Thyroid Gland.

The design of this gland, I believe to be to defend the brain from the morbid effects of all those causes which determine the blood into it, with unusual force.

My reasons for this belief are founded, 1st. upon its situation and structure. It is seated upon the anterior parts of the larynx, and is supplied with four large arteries, which return their blood by means of veins, without terminating in an excretory duct, or producing any thing like a secreted liquor.

2. Upon its larger size in women than in men. This provision was necessary to guard the female system from the influence of the more numerous causes of irritation and vexation of mind, and the more acute bodily diseases, to which they are exposed, than the male sex. The sensation known by the name of globus hystericus, appears to be produced by the diversion of excessive mental impressions from the brain to the thyroid gland. We often observe it to be considerably enlarged in hysterical paroxysms. A remarkable case of this kind, is taken notice of by Dr. Whyt, in his treatise upon nervous diseases. It is probably from the greater size, and more frequent excitement of the thyroid gland in women than in men, that the former are more subject to Bronchocele than the latter.

3. Upon the effect of certain exercises of the body and mind, upon the thyroid gland in its diseased state. Dr. Broadbelt relates in his inaugural dissertation upon bronchocele, published in Edinburgh, in the year 1794, that such of the inhabitants of Derbyshire, in England, as are afflicted with that disease, are subject to a pain in the gland, gland, and to an increase of its size when they are unusually excited by running or anger.

4. Upon the effect which disease in the thyroid gland, and its loss, have upon the brain. The bronchocele of the Cretins is generally accompanied with imbecility of mind; and Dr. Chapman informed me that Mr. Cooper, Surgeon of St. Thomas's Hospital, in London, had produced something like fatuity in several dogs, by extirpating this gland.

It is possible this gland may serve the additional purpose of an outlet, to undue impressions upon the lungs and windpipe, by an excess in the exercises of the voice and speech, and thereby defend those important parts of the body from rupture and disease.

I shall conclude this inquiry, into the functions of the spleen, liver, pancreas, and thyroid gland, by informing the reader, that I have yielded reluctantly to the solicitations of my pupils, and the advice of my medical friends, in offering it to the public in its present immature state. My best wishes are for an early examination, and a speedy refutation of the opinions contained in it, if they are erroneous; and if otherwise, that the inferences to which they lead, may be immediately applied to the practice of medicine.*

*Philadelphia, May* 13*th,* 1806.

---

### *To the Editors of the Medical and Phyſical Journal.*

GENTLEMEN,

HAVING found considerable advantage from the use of the seton after operations on the eyes, especially after couching; I hope the recommendation of it to the Faculty in those cases, may not be deemed intruding upon the sheets of your valuable Journal.

If my experience in many instances had not clearly convinced

---

* Since I have adopted, and taught the use I have assigned to the spleen, a former pupil of mine, copied from a French edition of one of Heister's works, by Dr. Senac, in the possession of a physician in a neighbouring state, and put into my hands a short note, in which the Doctor glances at the opinion I have delivered; but rejects it as chimerical. The peculiar ideas upon that subject, and all the others contained in the above inquiry, occurred to me, while I was employed in investigating the causes of the obstructions in the spleen and liver, which take place in madness.

